# The Consequences of Mixed-Species Malaria Parasite Co-Infections in Mice and Mosquitoes for Disease Severity, Parasite Fitness, and Transmission Success

**DOI:** 10.3389/fimmu.2019.03072

**Published:** 2020-01-22

**Authors:** Jianxia Tang, Thomas J. Templeton, Jun Cao, Richard Culleton

**Affiliations:** ^1^National Health Commission Key Laboratory of Parasitic Disease Control and Prevention, Jiangsu Provincial Key Laboratory on Parasite and Vector Control Technology, Jiangsu Institute of Parasitic Diseases, Wuxi, China; ^2^Malaria Unit, Department of Pathology, Institute of Tropical Medicine, Nagasaki University, Nagasaki, Japan; ^3^Department of Protozoology, Institute of Tropical Medicine, Nagasaki University, Nagasaki, Japan

**Keywords:** malaria, mixed-species, virulence, co-infections, *Plasmodium yoelii*, *Plasmodium vinckei*, *Plasmodium chabaudi*

## Abstract

The distributions of human malaria parasite species overlap in most malarious regions of the world, and co-infections involving two or more malaria parasite species are common. Little is known about the consequences of interactions between species during co-infection for disease severity and parasite transmission success. Anti-malarial interventions can have disproportionate effects on malaria parasite species and may locally differentially reduce the number of species in circulation. Thus, it is important to have a clearer understanding of how the interactions between species affect disease and transmission dynamics. Controlled competition experiments using human malaria parasites are impossible, and thus we assessed the consequences of mixed-species infections on parasite fitness, disease severity, and transmission success using the rodent malaria parasite species *Plasmodium chabaudi, Plasmodium yoelii*, and *Plasmodium vinckei*. We compared the fitness of individual species within single species and co-infections in mice. We also assessed the disease severity of single vs. mixed infections in mice by measuring mortality rates, anemia, and weight loss. Finally, we compared the transmission success of parasites in single or mixed species infections by quantifying oocyst development in *Anopheles stephensi* mosquitoes. We found that co-infections of *P. yoelii* with either *P. vinckei* or *P. chabaudi* led to a dramatic increase in infection virulence, with 100% mortality observed in mixed species infections, compared to no mortality for *P. yoelii* and *P. vinckei* single infections, and 40% mortality for *P. chabaudi* single infections. The increased mortality in the mixed infections was associated with an inability to clear parasitaemia, with the non-*P. yoelii* parasite species persisting at higher parasite densities than in single infections. *P. yoelii* growth was suppressed in all mixed infections compared to single infections. Transmissibility of *P. vinckei* and *P. chabaudi* to mosquitoes was also reduced in the presence of *P. yoelii* in co-infections compared to single infections. The increased virulence of co-infections containing *P. yoelii* (reticulocyte restricted) and *P. chabaudi* or *P. vinckei* (predominantly normocyte restricted) may be due to parasite cell tropism and/or immune modulation of the host. We explain the reduction in transmission success of species in co-infections in terms of inter-species gamete incompatibility.

## Introduction

Eight malaria parasite species are infectious to humans; namely, *Plasmodium falciparum, Plasmodium vivax, Plasmodium malariae, Plasmodium ovale wallikeri, Plasmodium ovale curtisii, Plasmodium knowlesi, Plasmodium cynomolgi*, and *Plasmodium simium*. The latter three species are parasites of non-human primates, but also cause zoonotic malaria in humans (1–3). In large parts of the tropical world the ranges of at least some of these species overlap, they are often vectored by the same mosquitoes (4), and mixed-species infections are common (5–7).

Mixed species infections of human malaria parasites are well-documented in natural (8–12) and experimental [e.g., (13, 14)] settings. They are studied regarding diagnosis (15–17), treatment (18), immune response (19), virulence (12, 20), transmission (21–23), and in discussions of public health policy (6). The virulence of malaria infection is also of interest in the context of co-infection with other pathogens, such as HIV and *Schistosoma* (24–26).

The consequences of mixed-species infections on malaria disease and parasite fitness are incompletely understood. There is conflicting evidence from laboratory and field studies regarding the capacity of mixed-species infections to exacerbate (27) or ameliorate (14) disease. Furthermore, the mechanisms underlying the interactions between parasite species in mixed infections are complicated and multi-factorial, possibly involving both within-host competition (28), and cross-immunity (29).

Mixed species and mixed strain *Plasmodium* infections have been studied in primate (30) and rodent malaria parasite models (27, 28, 31, 32), as these enable the study of all parasite lifecycle stages including those that occur in mosquitoes. The rodent model is enhanced by the availability of multiple rodent malaria parasite species; namely, *Plasmodium yoelii, Plasmodium berghei, Plasmodium vinckei*, and *Plasmodium chabaudi*. For *P. yoelii* there are additionally several parasite strains that differ in virulence following inoculation of mice (33).

Previous studies on the consequences of mixed species infections for disease pathology using the rodent malaria parasites in mice have yielded conflicting and varied results. Snounou et al. found that the virulence of mixed-species infections, as measured by host mortality, was reduced compared to that of single infections of the constituent species (34) whilst Ramiro et al. found the opposite effect (27).

Here we describe the results of a series of experiments utilizing multiple strains of the rodent malaria parasite species *P. yoelii, P. chabaudi*, and *P. vinckei*, in which mixed infections of various combinations of species and strains were established and studied in both mice and mosquitoes. These rodent malaria parasite species display important phenotypic differences, specifically in their red blood cell type tropism, that impact on disease progression. The consequences of mixed species infections for disease severity in both hosts, parasite fitness, and transmission capacity were analyzed.

## Materials and Methods

### Parasites, Mice and Mosquitoes

Four rodent malaria parasite strains, comprising three species, were used in these experiments; specifically, *P. chabaudi chabaudi* clone AJ, *P. chabaudi chabaudi* clone AS_ED_ (intermediate virulence, normocyte preference) (35), *P. yoelii* clone CU (non-virulent, reticulocyte restricted) (32), and *P. vinckei lentum* clone DS (non-virulent, normocyte preference) (36). These parasite lines were obtained from deep-frozen stocks kept at the University of Edinburgh (curated by Professors Richard Carter and David Walliker) and were originally isolated from thicket rats in Central Africa (35). Six-week-old female CBA mice, *Mus musculus*, were purchased from SLC Inc. (Shizuoka, Japan) and were used for all experiments. Mice were housed in a 12-h/12-h light/dark cycle at 24°C and fed with 0.05% para-aminobenzoic acid (PABA)-supplemented water to assist the growth of parasites. *Anopheles stephensi* mosquitoes were housed in a temperature- and humidity-controlled insectary at 23°C and 75% humidity. Mosquitoes used in the transmission experiments were maintained on 10% glucose solution supplemented with 0.05% PABA.

### DNA Extraction and Real Time Quantitative PCR (qPCR)

To determine the proportion of each species in mixed infections, quantitative real time PCR (qPCR) was used to measure copy numbers of the merozoite surface protein 1 gene (*msp1*). DNA was extracted from infected mouse tail blood and infected mosquito midguts using an EZI DNA Investigator Kit (GIAGQN) according to the manufacturer's instructions. Quantitative PCR was performed on an ABI 7500 real-time PCR machine using a Power SYBR Green kit (Applied Biosystems, UK). Primers were designed based on a species-specific region of *msp1*, as follows: *Py*CUmsp1F 5′CACCCTCAATAAACCCTGC-3′, *Py*CUmsp1R 5′-CGTGTACCAATACTTGAGTCAGAAC-3′; *Pv*DSmsp1F 5′-CAAGAAGCCTCACAACAAGAATCTA-3′, *Pv*DSmsp1R, 5′TGCTGGTTGGGCAGGTGCTGGA-3′, and *Pc*AJmsp1F 5′-GTACAAGAAGGAGCATCAGC-3′, *Pc*AJmsp1R 5′-GCGGGTTCTGTTGAGGCTCCT-3′. PCR assays were conducted on an AB7500 real-time PCR machine (Applied Biosystems, Japan) under the conditions: initial denaturation step of 50°C for 2 min, 95°C for 10 min, followed by 40 cycles of 95°C for 15 s and finally 61°C for 1 min. Copy numbers of *msp1* were quantified with reference to a standard curve generated from known numbers of plasmids containing the target sequence. As different parasite species have differing mean copy numbers of *msp1* per infected erythrocyte (due to different rates of DNA replication, differing numbers of merozoites per schizont, and differing propensities for multiple erythrocyte invasion), we normalized the proportion of each species in mixed infections by the copy numbers per infected erythrocyte calculated from single species infections. This methodology was also used to quantify the numbers of species-specific oocysts on the midguts of co-infected mosquitoes.

### Experiments Involving the Monitoring of Virulence in Mice

Eight experimental groups of five mice each were set up to measure the effects of co-infections of parasite species on mice and to compare the growth of species in single and mixed species infections. Three of these groups were inoculated via intravenous (IV) injection with parasite infected red blood cells (iRBCs) of a single *P. c. chabaudi* clone AJ (hereafter referred to as *Pc*AJ), *P. c. chabaudi* AS_ED_ (*Pc*AS_ED_), *P. y. yoelii* clone CU (*Py*CU) or *P. v. lentum* clone DS (*Pv*DS). The remaining four received mixtures of two species in equal numbers (*Pc*AJ+*Py*CU, *Py*CU+*Pc*AS_ED_*, Py*CU+*Pv*DS and *Pc*AJ+*Pv*DS). Inocula were diluted in a solution of 50% fetal calf Serum (FCS) and 50% Ringer's solution (27 mM KCl, 27 mM CaCl_2_, 0.15 M NaCl). Mice infected with single parasite species received 10^6^ iRBCs, and co-infected mice received 10^6^ of each component parasite species. It has been shown that a 2-fold difference in parasite numbers has a negligible effect on parasite dynamics and virulence (37).

Virulence was determined using the parameters of mortality, weight loss, and reduction in erythrocyte density, and was measured daily up to day 30 post-inoculation. Erythrocyte densities were counted using a Coulter Counter (Backman coulter, Florida) from 1:40,000 dilution of 2 μl whole blood sampled from tails in Isoton solution (Beckman coulter, Florida). Giemsa's solution stained thin blood smears from tail vein blood were monitored for parasitaemia for 30 days post-inoculation to assess the parasite replication rate. Whole blood samples (10 μl) were collected daily from day 1 to 30 into citrate saline, centrifuged briefly, and the erythrocyte pellet stored at −80°C prior to DNA extraction using an EZ1 DNA Investigator Kit (QIAGEN, Japan) and an EZ1 BioRobot (QIAGEN, Japan). Species specific qPCR based on the *msp1* gene was used to measure the proportions of each parasite in the mixed infections (32, 38). All experiments were performed twice.

### Mosquito Transmission Experiments: Estimation of Mosquito Fitness and Parasite Species Transmission Capacity

Groups of mice were infected with single and mixed species infections of *Py*CU, *Pv*DS, and *Pc*AJ parasites (total six groups, each of five mice). On days 3 and 5 post-inoculation, individual groups of mosquitoes (*n* = 40 mosquitoes per group; 5–7 days post emergence from pupae) were fed on individual mice. Immediately following the feed, 20 mosquitoes from each group were pooled by mouse group into 12 cages, and egg bowls added 2 days later to allow the collection of eggs. These groups were monitored for longevity by counting dead mosquitoes daily up to day 60, and the numbers of larvae produced per mosquito were counted at day 5 post-hatching. Seven days later, 20 mosquitoes were removed from each group (57 groups total, as the number of mice fed from group *Pv*DS and *Pc*AJ + *Pv*DS was reduced to three and four, respectively for the day 5 feed) and the midgut oocyst burden recorded following dissection. Dissected midguts were stored at −80°C prior to DNA extraction for species proportion analysis by qPCR.

### Statistical Analyses

All graphs were generated using GraphPad Prism 6 (GraphPad software Inc, USA). Comparison of survival curves was carried out using Log-rank (Mantel-Cox) tests. Multiple *t*-tests, corrected for multiple comparisons using the Holm-Sidak method, were used for comparing parasitaemia, erythrocyte density, weight loss, and parasite density of single and mixed infection in mice at all days during infection. Mann Whitney tests were carried out for cumulative parasite density, mosquito infection, and analysis of oocysts per gravid mosquito. *P*-values of below 0.05 were considered significant.

## Results

### Mixed Species Parasite Infections Involving a Reticulocyte Specialist and a Normocyte Specialist Are More Virulent and Cause Greater Host Mortality Than Single Species Infections in Mice

**Figure 1:**
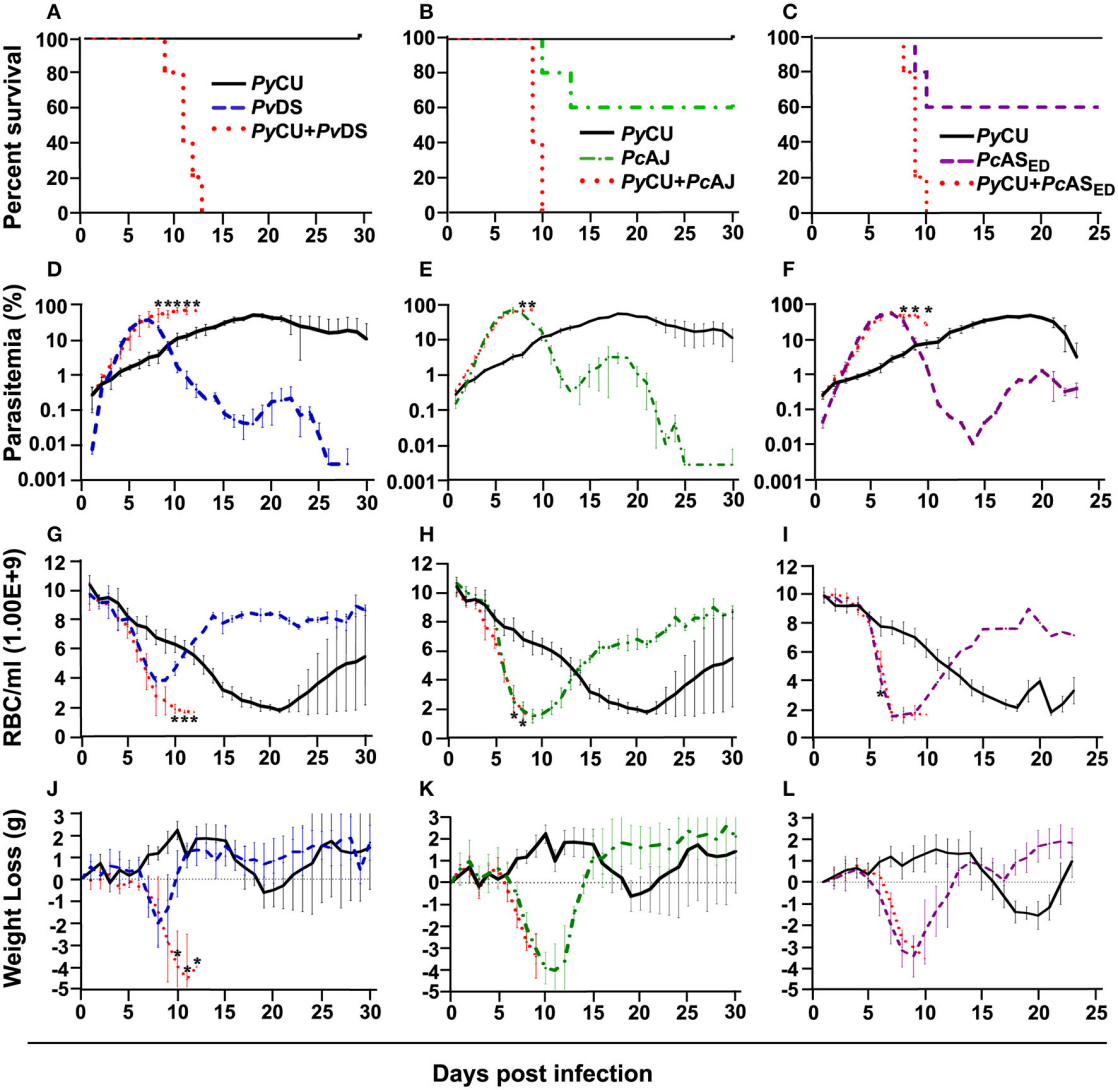
Percent survival **(A–C)**, parasitaemia **(D–F)**, erythrocyte density **(G–I)**, and weight loss **(J–L)** of mice infected with *Plasmodium yoelii* CU (black), *Plasmodium vinckei* DS (blue), *Plasmodium chabaudi* AJ (green), and *Plasmodium chabaudi* AS_ED_ (purple) species in single and mixed infections (red). Mice were inoculated via intravenous injection of 1 × 10^6^ infected red blood cells of either single or mixed species of the above parasites and followed for 25–30 days to determine mortality, live-body weight, erythrocyte density, and parasitaemia. Data points indicate the mean value for five mice in each experimental group and error bars indicate the standard error of the mean (SEM). An asterisk represents statistically significant differences between mixed infections compared with both single infections (Multiple *t*-tests, with the assumption that all rows are sampled from populations with the same scatter and corrected for multiple comparisons using the Holm-Sidak method). Parasitaemias of *Py*CU and *Pv*DS in mixed-infections were significantly different between days 8 and 12 post infection (PI) compared with the same strains in single infections **(D)**; *Py*CU and *Pc*AJ mixed-infections were significantly different to single infections of the same strains on days 8 and 9 PI **(E)**; and *Py*CU and *Pc*AS_ED_ mixed-infections were significantly different from single infections between days 8 and 10 PI **(F)**. Erythrocyte densities of *Py*CU and *Pv*DS mixed-infections were significantly different from single infections on day 10 to 12 PI **(G)**; *Py*CU and *Pc*AJ mixed-infections were significantly different on days 7 and 8 PI **(H)**; while mixed infections of *Py*CU and *Pc*AS_ED_ were significantly different from single infections only on day 6 PI **(I)**. Mice infected with *Py*CU+*Pv*DS mixed-infections lost significantly more weight compared to single infections from day 10 to 12 **(J)**. Mice in the groups infected with mixed infections of *Py*CU+*Pc*AJ suffered from significantly reduced erythrocyte density compared to mice in single infection groups (Two-way RM ANOVA measured mixed effects model, *P* = 0.043, *F* = 5.7, DFn = 1, DFd = 8). Detailed statistical values relating to significance are given in Supporting Table 1. Experiments were repeated twice, data is from one representative experiment.

Infection parameters for single and mixed infections involving *Py*CU (reticulocyte restricted) and either *Pv*DS, *Pc*AJ, or *Pc*AS_ED_ (normocyte preference) are summarized in Table 1. *Py*CU and *Pv*DS are not lethal in single species infections and only the intermediately virulent species *P. chabaudi* (*Pc*AJ and *Pc*AS_ED_) caused death of mice in single infections, with 40% mortality occurring between days 9 and 13 post-infection (PI) for both *Pc* strains. In contrast, mixed-species co-infections of *Py*CU with either *Pv*DS, *Pc*AJ, or *Pc*AS_ED_ resulted in highly virulent infections with 100% mortality (Figures 1A–C).

**Table 1:** Infection parameters for single and mixed species infections of *Plasmodium yoelii* CU with *Plasmodium vinckei* DS, *Plasmodium chabaudi* AJ, and *Plasmodium chabaudi* AS_ED_.

**Strain**	***Py*CU**	***Pv*DS**	***Pc*AJ**	***Pc*AS_**ED**_**	***Py*CU + *Pv*DS**	***Py*CU + *Pc*AJ**	***Py*CU + *Pc*AS_**ED**_**
**RBC invasion preference**	**Reticulocytes**	**Normocytes**	**Normocytes and reticulocytes**	**Normocytes and reticulocytes**	/	/	/
Peak Parasitaemia % (day pi)	52.57 ± 1.89 (18–19, 25)	36.44 ± 0.54 (6–7)	63.85 ± 4.94 (7)	59.91 ± 1.03 (6, 7)	75.78 ± 1.72 (8, 10–11)	59.96 ± 3.56 (7–8)	64.32 ± 3.43 (7, 9)
Cumulative parasitaemia	544.74 ± 80.00	127.94 ± 6.32	245.55 ± 14.83	190.21 ± 7.49	281.52 ± 26.66	218.39 ± 16.87	188.53 ± 9.45
Mortality (day pi)	0%	0%	40% (10, 13)	40% (9, 10)	100% (9, 11, 12)	100% (9, 10)	100% (9–11)
Max. weight loss, g (day pi)	1.26 ± 0.44 (19–21, 27)	2.49 ± 1.00 (8, 10)	4.06 ± 0.54 (9–13)	3.54 ± 0.38 (8, 9)	4.26 ± 0.66 (8, 9–12)	2.93 ± 0.30 (8–9)	2.92 ± 0.18 (8, 10)
Min. RBC count, RBC/ml (day pi)	1.61 × 10^9^ ± 0.13 × 10^9^ (21, 26, 28)	3.61 × 10^9^ ± 0.05 × 10^9^ (8, 9)	1.51 × 10^9^ ± 0.21 × 10^9^ (9)	1.41 × 10^9^ ± 0.13 × 10^9^ (7, 8)	1.60 × 10^9^ ± 0.04 × 10^9^ (8–11)	1.65 × 10^9^ ± 0.22 × 10^9^ (8, 9)	1.24 × 10 ^9^ ± 0.08 × 10^9^ (7, 8)

Mixed species infections resulted in higher parasitaemia than either of their constituent species in single infections (Figure 1D) and peak parasitaemia occurred on the same day PI as the more virulent of the constituent species; except for *Py*CU + *Pv*DS in which peak parasitaemia occurred between days 8 and 11, compared to the *Pv*DS single infection, in which peak parasitaemia occurred at days 6–7 (Figures 1D–F). Host mortality in mixed species infections occurred at peak parasitaemia and was presumably caused by anemia resulting from an inability to clear parasites from the blood.

During the latter stages of the infection mixed species infections involving *Pv*DS and *Py*CU resulted in lower erythrocyte densities (Figures 1G–I) and greater weight loss (Figures 1J–L) compared to either of the constituent species in single infections. This increased pathology was linked to the inability of mice to control parasitaemia.

### Mixed Species Parasite Infections Involving Two Normocyte Specialists Result in Protracted Parasitaemia, but Not Increased Virulence

**Figure 2:**
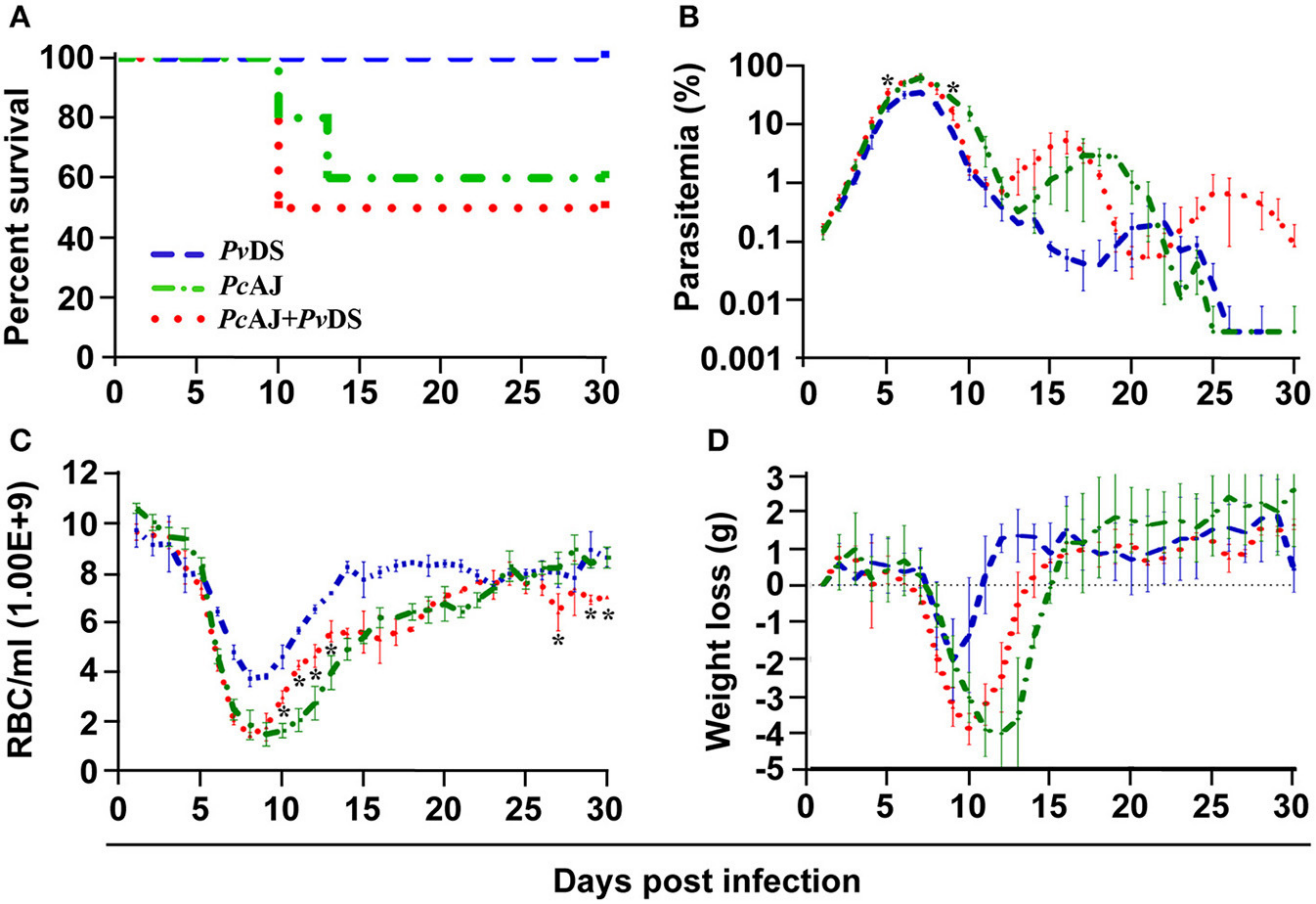
Percentage survival **(A)**, parasitaemia **(B)**, erythrocyte density **(C)**, and weight loss **(D)** of infected mice with *Plasmodium vinckei* DS (blue), *Plasmodium chabaudi* AJ (green) single or mixed infections (red). Mice were inoculated via intravenous injection of 1 x 10^6^ infected red blood cells of either single or mixed species of the above parasites and followed for 30 days to determine mortality, live-body weight, erythrocyte density, and parasitaemia. Data points indicate the mean value for mice of each experimental group and error bars indicate the SEM. An asterisk represents statistically significant difference of mixed infections compared with both single infections (Multiple *t*-tests, with the assumption that all rows are sampled from populations with the same scatter and corrected for multiple comparisons using the Holm-Sidak method). Mice infected with mixed-infections developed statistically significantly higher parasitaemia on days 5 and 9 PI compared with both single infections **(B)**. Mice infected with mixed-infections had significantly lower erythrocyte densities on days 10 to 13, 27, 29, and 30 PI compared with both single infections **(C)**. Considering the entire time course of the infections, the parasitaemia of mice infected with single infections of *Pv*DS were significantly lower than those of mice infected with *Pv*DS + *Pc*AJ mixed-infections (two-way RM ANOVA measured mixed effects model, *F* = 218.8, DFn = 1, DFd = 3, *P* = 0.0007). The erythrocyte density of mice infected with *Pv*DS + *Pc*AJ mixed-infections was significantly lower than those infected with single infections of *Pv*DS (two-way RM ANOVA measured mixed effects model, *F* = 123, DFn = 1, DFd = 3, *P* = 0.0016). Detailed statistical values relating to significance are given in Supporting Table 1. Experiments were repeated twice, data is from one representative experiment.

*Plasmodium vinckei* DS and *P. chabaudi* AJ are both normocyte invading parasites. *Pc*AJ is of moderate virulence, causing rapid and severe anemia and weight loss during the first 10 days of infection when parasitaemia rises, and results in 40% host mortality (Table 2 and Figure 2A). *Pv*DS is a much less virulent parasite, causing less severe weight loss and milder anemia, and is never lethal (Table 2 and Figure 2A). The combination of these two parasites in a mixed infection results in 50% host mortality, and a pathology consistent with that of *Pc*AJ, the more virulent of the two species (Table 2 and Figure 2A). However, the *Pc*AJ+*Pv*DS infection results in three distinct parasitaemia peaks, compared to the two peaks produced by the single species, and parasitaemias persisted up to the last day of the experiment (day 30), compared to clearance by day 26 in the single species infection (Figure 2B). The *Pc*AJ+*Pv*DS infection also displayed a sharper decline in parasitaemia following the first peak (Figure 2B), and this was associated with a quicker recovery from anemia and weight loss between days 10 and 15 (Figures 2C,D).

**Table 2:** Infection parameters for single and mixed species infections of *Plasmodium vinckei* DS and *Plasmodium chabaudi* AJ.

**Strain**	***Pv*DS**	***Pc*AJ**	***Pc*AJ + *Pv*DS**
**RBC invasion preference**	**Normocytes**	**Normocytes and reticulocytes**	**/**
Peak parasitaemia % (day pi)	36.44 ± 0.54 (6–7)	63.85 ± 4.94 (7)	65.67 ± 2.28 (6–7)
Cumulative parasitaemia	127.94 ± 6.32	245.55 ± 14.83	240.15 ± 6.07
Mortality (day pi)	0%	40% (10, 13)	50% (10)
Max. weight loss, g (day pi)	2.49 ± 1.00 (8, 10)	4.06 ± 0.54 (9–13)	3.98 ± 0.12 (9–10)
Min. RBC count, RBC/ml (day pi)	3.61 × 10^9^ ± 0.05 × 10^9^ (8, 9)	1.51 × 10^9^ ± 0.21 × 10^9^ (9)	1.46 × 10^9^ ± 0.05 × 10^9^ (8, 9)

### Co-Infection of *P. yoelii* With Either *P. chabaudi* or *P. vinckei* Results in Reduced Parasite Density of *P. yoelii*, and Protracted Peak Parasitaemia of *P. chabaudi* and *P. vinckei*

To understand how mixed parasite species infection influences the fitness of the species involved, we measured the parasite density (numbers of parasites per ml of mouse blood) through time of individual species in single and mixed infections. The relative proportions of each species within mixed infections were measured at 24-h intervals by species specific qPCR.

**Figure 3:**
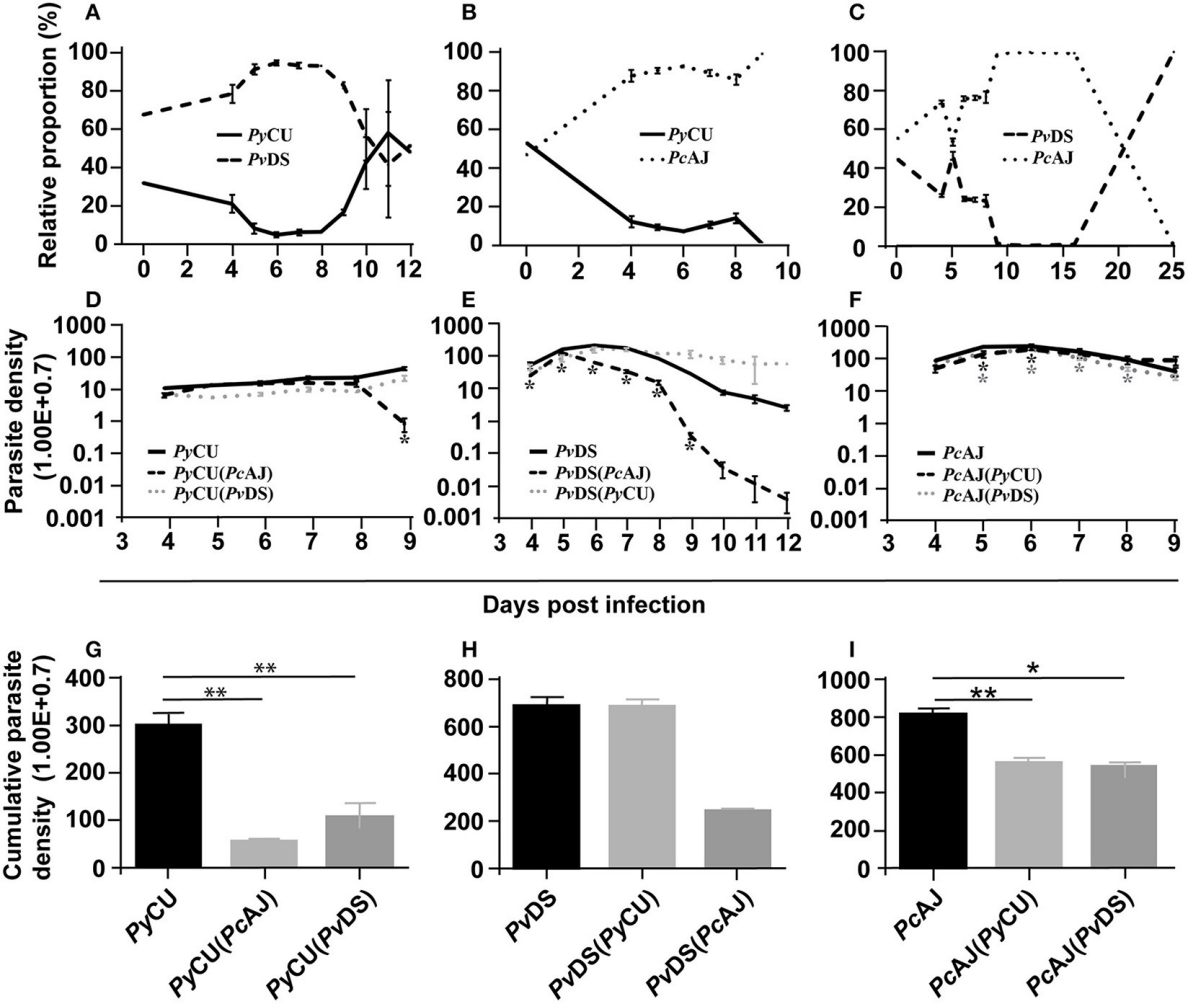
The relative proportions of *Plasmodium yoelii* CU, *Plasmodium vinckei* DS, and *Plasmodium chabaudi* AJ in mixed infections **(A–C)**, the parasite density of each species in single and mixed infections **(D–F)**, and cumulative parasite density of each species in either single or mixed infections **(G–I)**. The relative proportion of each species in combination with each other **(A–C)** was measured by qPCR quantification using primers specific to a region of the *msp1* gene of each species. Copy numbers of parasite *msp1* were quantified with reference to a standard curve generated from known numbers of plasmids containing the same gene sequences. The average copy numbers per iRBC were generated by copy numbers and parasite densities of each single species infections on day 6 PI. Data points indicate the mean value for 3–5 mice in each experimental group and error bars indicate the SEM. The parasite densities (number of blood stage parasites per mL blood) are shown in **(D–F)**. **(D)** Shows the parasite densities of *Py*CU single infection or in mixed infection with *Pv*DS or *Pc*AJ. Parasite densities of PvDS and PcAJ are given in **(E,F)**, respectively. An asterisk represents significant differences (*P* < 0.01) in parasite density in single infections compared with mixed-infections. Cumulative parasite densities are shown in **(G–I)**. The cumulative parasite density of *Py*CU in single infections were significantly higher than *Py*CU in mixed-infections with *Py*AJ and *Pv*DS **(G)**. Similarly, the cumulative parasite density of *Pc*AJ in single infections was significantly higher than in mixed infections with *Py*CU or *Pv*DS **(I)**. Detailed statistical values relating to significance are given in Supporting Table 1. Experiments were repeated twice, data is from one representative experiment.

In mixed infections composed of *Py*CU and *Pv*DS*, Pv*DS dominated the infection from days 4 to 10 PI (Figure 3A), at which point *Py*CU became dominant. There was an increase in the proportion of *Py*CU in the infection from day 8 (7%) until host mortality at day 12 PI (50%). Analysis of species-specific parasite density in this co-infection revealed that *Py*CU was suppressed throughout the infection, while the growth of *Pv*DS was enhanced (Figures 3D,E).

This enhancement occurred during the latter stages of the infection (days 8–12), the time point at which *Pv*DS is cleared during single infections. This suggests that the presence of *Py*CU, whose growth in a co-infection does not differ significantly from that observed in a single species infection, facilitates the persistence of *Pv*DS for an extended period after which it would normally be cleared. This inability to clear *Pv*DS, combined with the standard increase in *Py*CU parasitaemia, leads to hyper-parasitaemia with severe anemia in co-infected mice, and results in host death.

Enhancement of the parasite density of the normocyte-restricted parasite species was also observed in the latter stages of mixed species infections composed of *Py*CU and *Pc*AJ. In this case, *Pc*AJ dominates *Py*CU throughout the infection, with complete exclusion of the latter species observed at the end of the co-infection (Figure 3B). Mice died at days 9 and 10 pi, at which point the parasite density of *Py*CU was significantly suppressed compared to single infections (Figure 3D). In contrast, there was little difference in the parasite density of *Pc*AJ in a mixed infection with *Py*CU compared to a single infection during the first 8 days of the co-infection. However, as seen in the *Pv*DS+*Py*CU infection (Figure 3E), the usual reduction in parasitaemia observed at day 8 in *Pc*AJ single infections was not observed in co-infections with *Py*CU (Figure 3F), suggesting again that presence of *Py*CU in a co-infection impairs the ability of the host to control the growth of the normocyte-invading parasite species.

Cumulative parasite densities were calculated for each parasite species in single and mixed infections as proxy measures of parasite productivity throughout the infection. There was a dramatic reduction in cumulative parasite density for *Py*CU in the mixed infections with both *Pc*AJ and *Pv*DS (Figure 3G). In the mixed infections involving *Pc*AJ and *Pv*DS, both of which preferentially invade normocytes, there was a dramatic reduction in cumulative parasite density for *Pv*DS throughout the co-infection compared to single infection (Figure 3H). There was also a slight reduction in cumulative parasite density of *Pc*AJ (Figure 3I), a reflection of lower productivity during the latter stages of the infection. *Pc*AJ is the more virulent of the two species and dominates the co-infection between days 5 and 15, when no *Pv*DS could be detected by qPCR. However, *Pv*DS resurges at day 15, and competitively excludes *Pc*AJ by day 25.

### Pre-exposure of Mice to *P. yoelii* Does Not Enhance the Virulence of *P. vinckei* Infection

**Figure 4:**
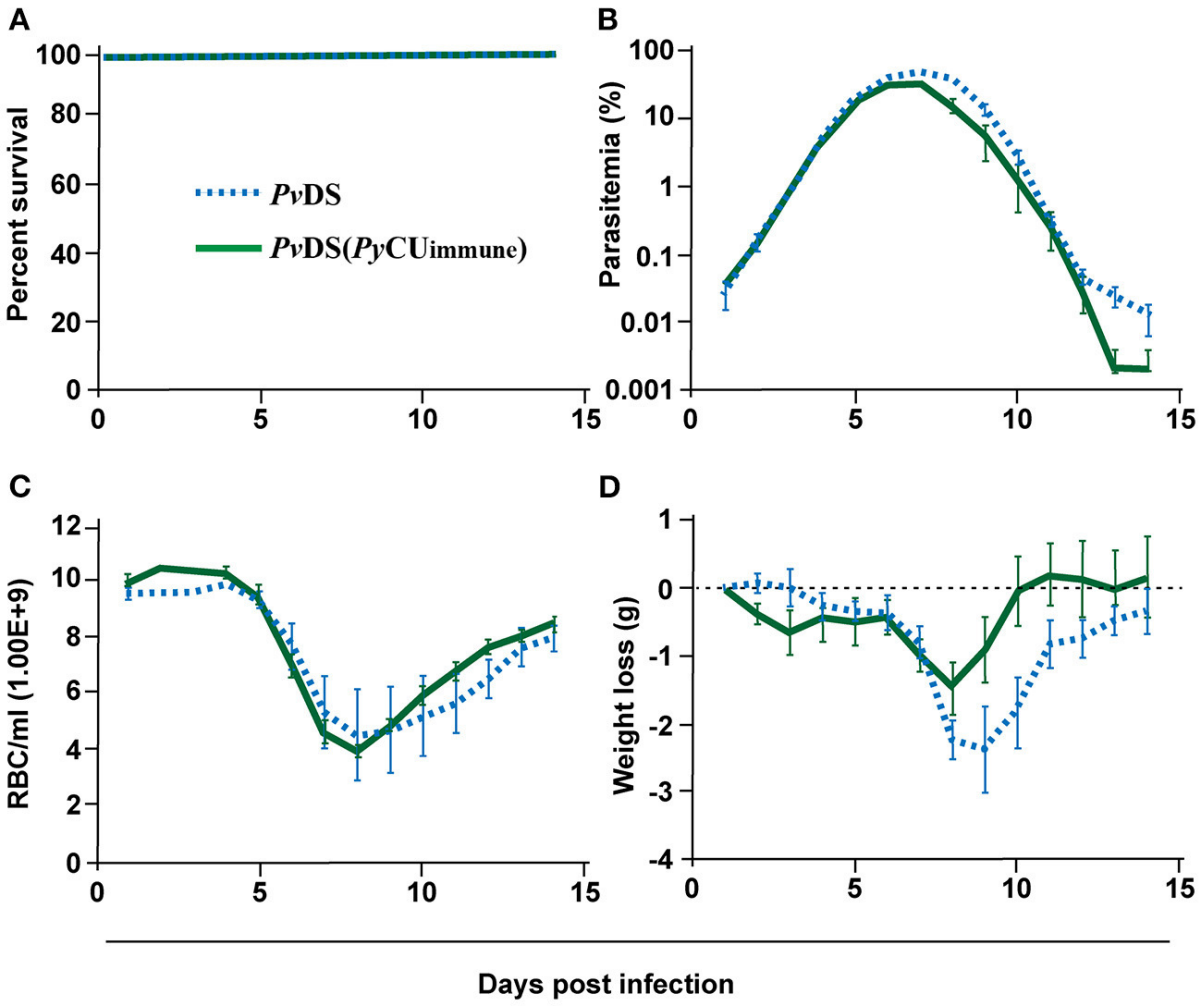
Percentage survival **(A)**, parasitaemia **(B)**, erythrocyte density **(C)**, and weight loss **(D)** of mice infected with *Plasmodium vinckei* DS (*Pv*DS). Mice in the *Pv*DS (*Py*CU immune) group were inoculated intravenously (IV) with 1 × 10^6^ infected red blood cells (iRBCs) of *Py*CU, treated 8 days later with mefloquine for 5 days, and then 15 days later intravenously challenged with 1 × 10^6^ *Pv*DS iRBCs. Data points indicate the mean value for 5 mice in each experimental group and error bars indicate the SEM. Detailed statistical values relating to significance are given in Supporting Table 1.

As the presence of *P. yoelii* in mixed infections with either *P. chabaudi* or *P. vinckei* results in an inability to clear the latter two species, resulting in host death, we wondered whether an immune response specific to *P. yoelii* could adversely affect the establishment of an effective immune response against *P. chabaudi* or *P. vinckei*. To test this, we pre-immunized mice with *Py*CU parasites by exposure to the parasite for 8 days followed by clearance with the anti-schizontal drug mefloquine (MF). Two weeks later, when all MF had cleared from the host, mice were challenged with *Pv*DS. In contrast to the patterns observed in *Py*Cu+*Pv*DS co-infections, there was no evidence of increased virulence of *Pv*DS infections in mice pre-exposed to *Py*CU (Figure 4A), with no significant enhancement of parasite density (Figure 4B), anemia (Figure 4C), or weight loss (Figure 4D) occurring at any stage during the infection.

### The Increased Virulence of Mixed Species Infections of *P. yoelii* and *P. vinckei* Is Abrogated When *P. vinckei* Is Added to an Established *P. yoelii* Infection

**Figure 5:**
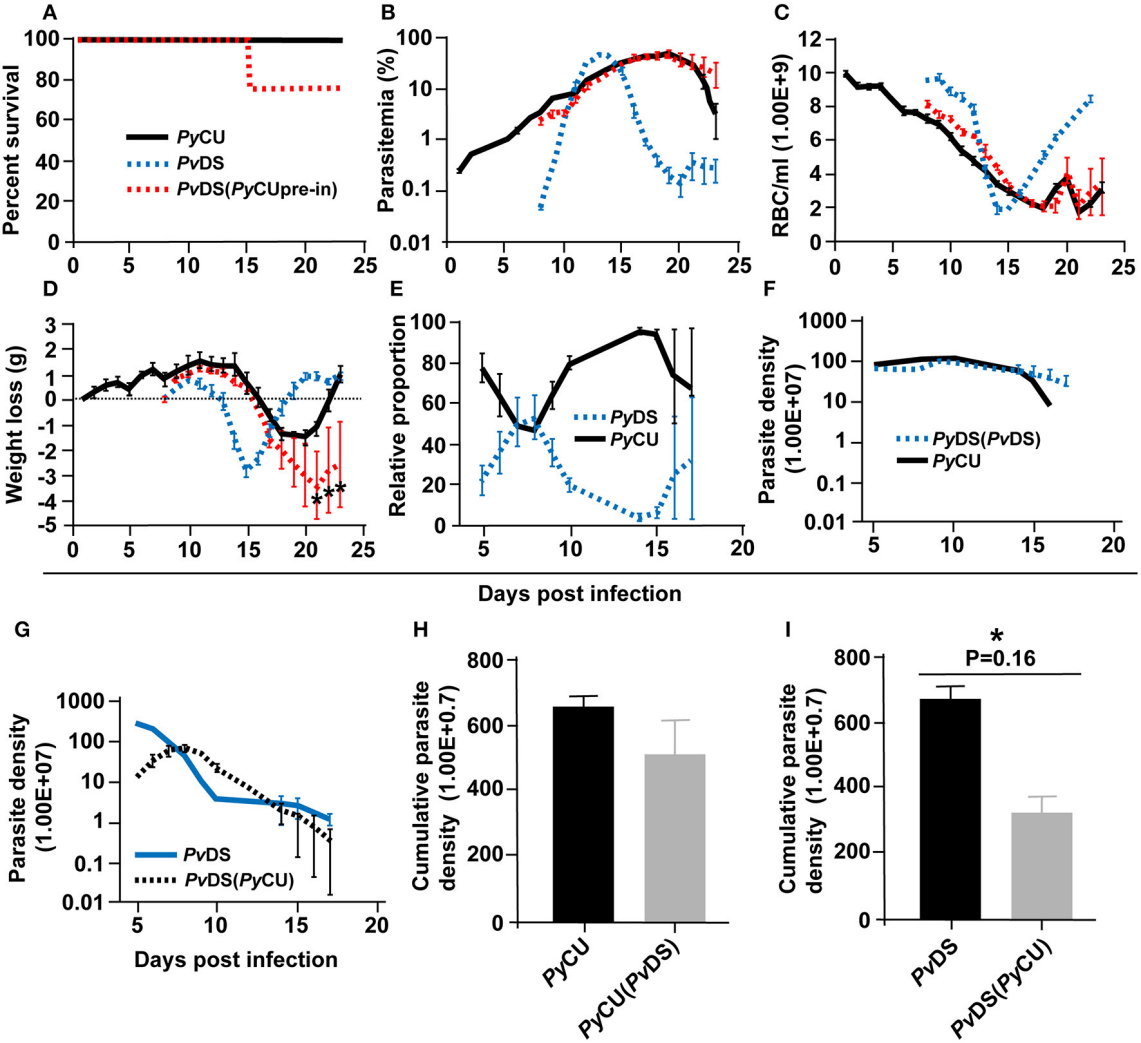
Percentage survival **(A)**, parasitaemia **(B)**, erythrocyte density **(C)**, and weight loss **(D)** of mice infected with *Plasmodium yoelii* CU (*Py*CU) and *Plasmodium vinckei* DS (*Pv*DS) in single and mixed infections. Mice in the mixed-infection group were inoculated intravenously (IV) with 1 × 10^6^ *Py*CU infected red blood cells (iRBCs) 7 days prior to IV inoculation with 1 × 10^6^ *Pv*DS iRBCs. Data points indicate the mean value for 4–5 mice in each experimental group and error bars indicate the SEM. Asterisks indicate statistically significant differences between mice with mixed-species infections compared with both single infections (Multiple *t*-tests, with the assumption that all rows are sampled from populations with the same scatter and corrected for multiple comparisons using the Holm-Sidak method). Mice infected with mixed-infections lost significantly more weight during the latter stages of the infection than mice infected with single species infections (days 21–23 PI, **D**). The relative proportion of each species in mixed infections **(E)** was measured by qPCR quantification of the *msp1* gene. The average copy number per iRBC was generated with reference to copy numbers and parasite densities of each single species infections on day 6 PI. The parasite densities of *Py*CU and *Pv*DS in single or mixed infections are shown in **(F,G)**. A Mann Whitney test shows that the cumulative parasite density of *Pv*DS in single infections is significantly higher than that of *Pv*DS in a mixed infection with *Py*CU **(I)**, whereas that of *Py*CU is unaffected when in a mixed infection with *Pv*DS **(H)**. Detailed statistical values relating to significance are given in Supporting Table 1.

When both *Py*Cu and *Pv*DS are inoculated into mice contemporaneously, the resulting co-infection is consistently lethal, in contrast to the zero-mortality associated with the constituent single species infections. This lethality results from the inability of mice to clear the *Pv*DS parasites from the circulation following peak parasitaemia. To determine if increased virulence is dependent on the timing of the introduction of the co-infecting species, we first inoculated mice with *Py*CU and introduced *Pv*DS 7 days later. The co-infection caused 25% mortality, compared to no mortality in single species infections (Figure 5A). In this case, the co-infection parasitaemia did not differ significantly from that of a single infection of *Py*CU for most of the infection duration, except for the last 2 days of sampling (days 22 and 23), when the parasitaemia was higher in the co-infection (Figure 5B). This increased parasitaemia toward the latter stages of the infection did not result in lower erythrocyte density (Figure 5C); however, it did cause significantly greater weight loss in co-infected animals during this period (Figure 5D).

Measuring the relative proportions and parasite densities of the constitute species in the co-infection and comparing them to single infections revealed that *Py*CU dominates the infection over *Pv*DS, excepting days 7 and 8 (Figure 5E). In contrast to the situation observed with the simultaneous inoculation of the two species, there was no significant reduction in the parasite density of *Py*CU, but there was a reduction in the parasite density of *Pv*DS when *Py*Cu was inoculated 1 week prior to *Pv*DS (Figures 5F–I).

### The Consequences of Mixed Species Infections in the Mosquito Vector

We additionally sought to describe the impact of mixed species infections on transmission to mosquitoes. Specifically, we fed *Anopheles stephensi* mosquitoes on mice with single or mixed species infections and measured: (i) the proportion subsequently infected, and (ii) the severity of this infection (number of oocysts); (iii) the longevity of infected mosquitoes; and (iv) the number of larvae they produced following a blood meal. Finally, we compared the transmission success, defined as the average number of oocysts produced per blood fed mosquito, of each parasite species in mixed or single infections.

### Mixed Species Infections Do Not Result in Significantly Different Infection Parameters in Mosquitoes

**Figure 6:**
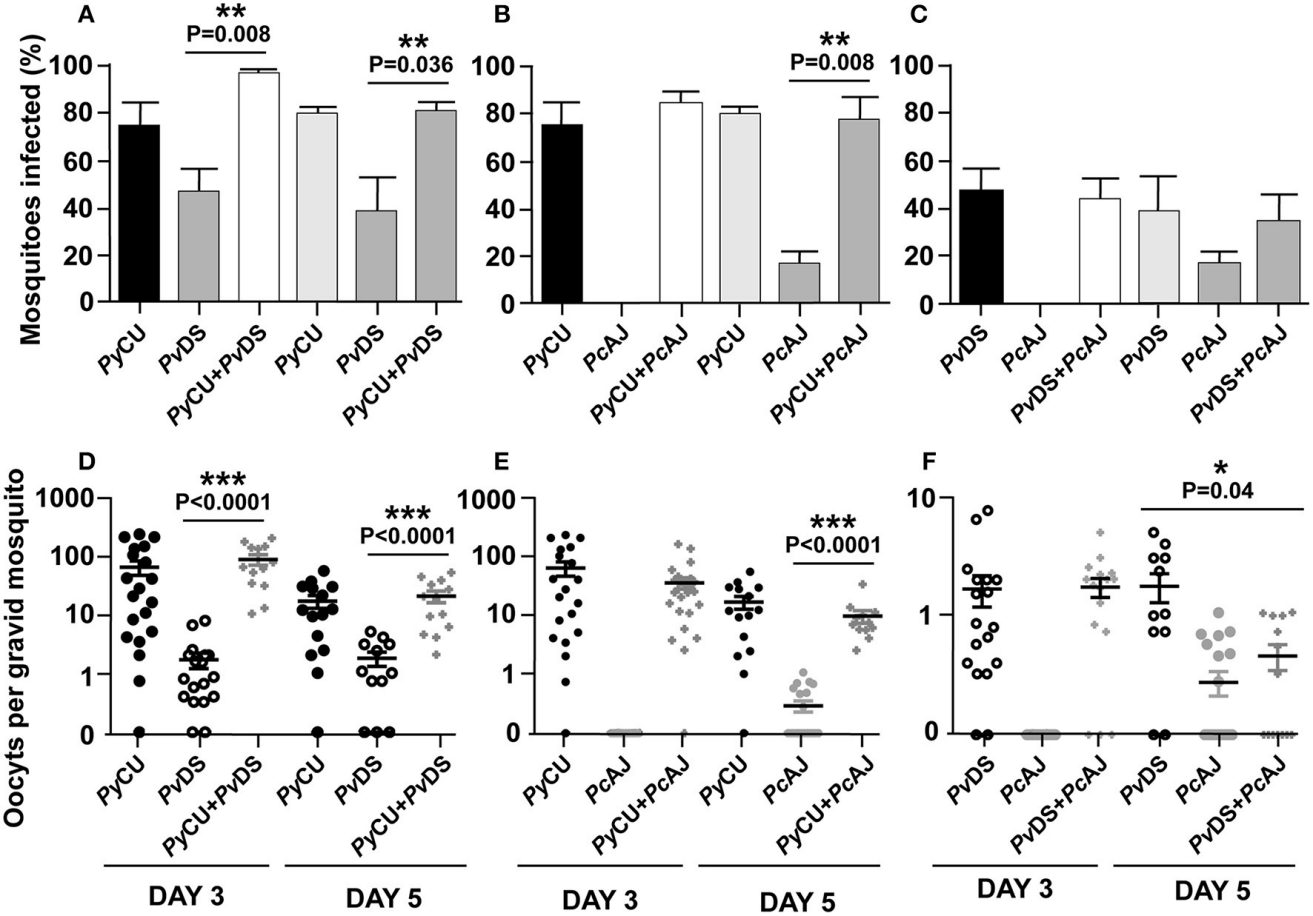
Transmission capacity of Plasmodium yoelii CU (PyCU), Plasmodium vinckei DS (PvDS), and Plasmodium chabaudi AJ (PcAJ) in single or mixed infections. The mean percentage of mosquitoes infected with oocysts were calculated following feeding on PyCU, PvDS, and PcAJ either in single or mixed infections in mice on days 3 and 5 PI **(A–C)**. The numbers of oocysts per gravid mosquito are given for all groups **(D–F)**. Only gravid mosquitoes were considered blood-fed and included in the analysis. Statistical analysis was performed using Mann Whitney tests. Detailed statistical values relating to significance are given in Supporting Table 1.

To determine whether mixed species infections result in altered mosquito infectivity rates and infection loads compared to single species infections, mosquitoes were fed on anesthetized mice infected with single, or mixed infections of *Pc*CU, *Pv*DS, and *Pc*AJ. As these species differ in the timing of their gametocyte production, with *Py*CU at its most infectious to mosquitoes on day 3 PI and *Pc*AJ and *Pv*DS more infectious on day 5 pi, we conducted mosquito feeds on both these days. *Py*Cu was the most infective single species on day 3 and 5, followed by *Pv*DS and finally *Pc*AJ (Figures 6A–C). Mixed species infections did not result in higher proportions of mosquitoes being infected than the most infective constituent species in a single infection (Figures 6A–C). Similarly, in co-infections containing the highly infectious *Py*CU species, the oocyst burdens of mixed species infections were not significantly different from that of *Py*CU in mosquitoes fed on mixed infections on either day 3 or 5 PI (Figures 6D,E). Mixed species infections of *Pc*AJ and *Pv*DS resulted in significantly lower oocyst burdens than the most infectious constituent single species (*Pv*DS), but only in mosquitoes fed on day 5 of the infection (Figure 6F).

### Co-Infections of Malaria Parasite Species Do Not Adversely Affect Mosquito Longevity or Capacity to Produce Larvae

**Figure 7:**
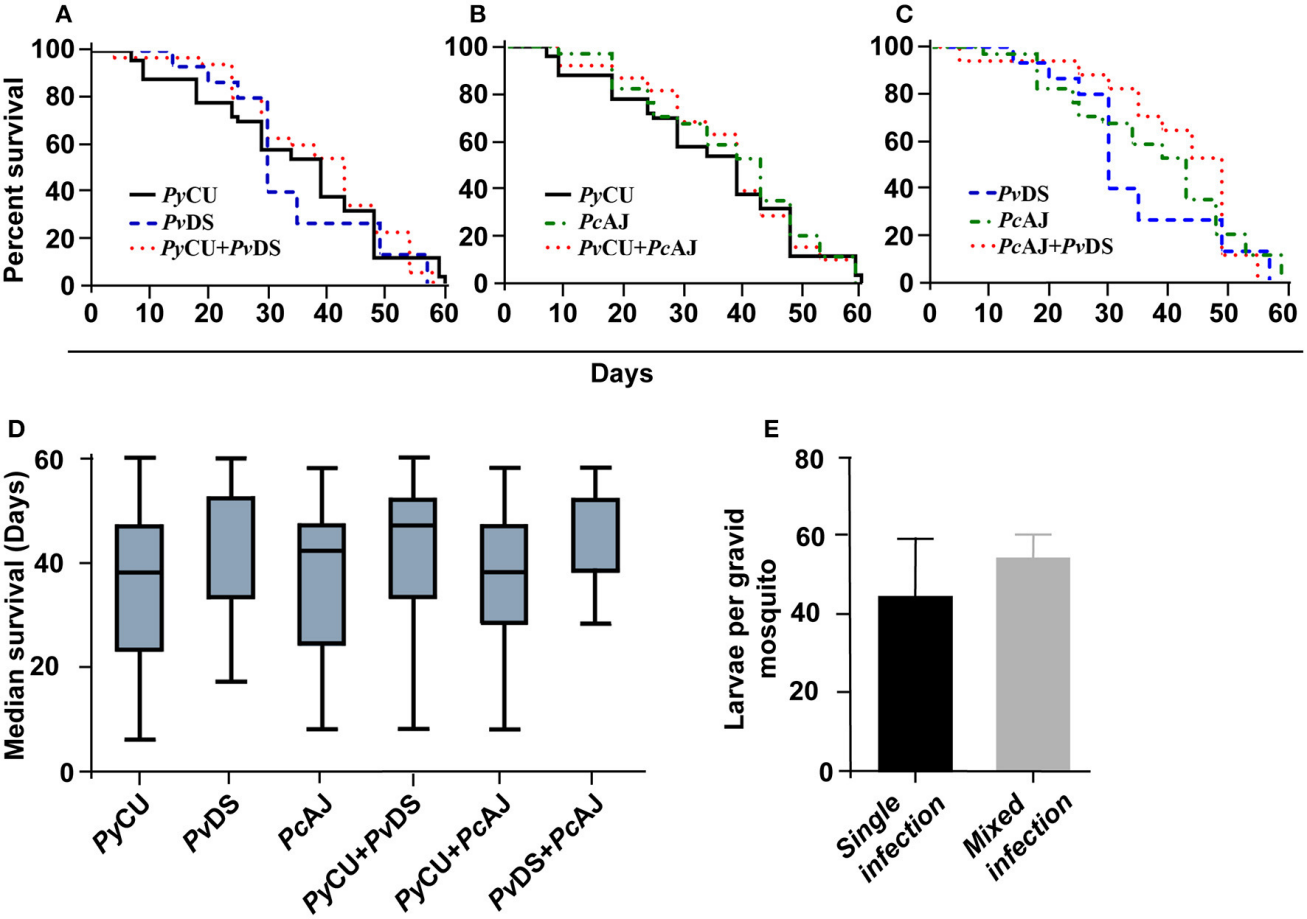
Survival **(A–D)** and larvae per mosquito **(E)** of mosquitoes infected with single and mixed species infections. Mosquito survival curves, infected with either single species or mixed infection of *Plasmodium yoelii* CU, *Plasmodium vinckei* DS, and *Plasmodium chabaudi* AJ are shown in **(A–C)**. Longevity of infected mosquitoes was observed until day 60 after the blood meal and the number of dead mosquitoes were recorded every 5 days. Boxplots indicate median survival and first and third quartiles, and whiskers are the same quartiles ± (1.5 × interquartile range) **(D)**. The numbers of larvae from infected mosquitoes with single or mixed infections were recorded and no significant difference was observed. Detailed statistical values relating to significance are given in Supporting Table 1.

To ascertain whether mixed species infections of mosquitoes were more virulent than single species infections, we measured longevity and larvae production in mosquitoes fed on single or mixed infections. In accordance with the observation that mixed species infections did not result in higher burdens of infection, mosquitoes infected with two parasite species in a co-infection did not display reduced longevity (Figures 7A–C), median survival time (Figure 7D), or reduced fitness (measured as the number of larvae produced per blood-fed mosquito) (Figure 7E).

### Mixed Species Infections Can Affect the Transmission Capacity of the Constituent Species

To determine whether mixed infections can affect the transmission capacity of constituent species, the relative proportion of each species in mixed infections in mosquitoes was measured using qPCR on DNA extracted from mosquitoes with known oocyst numbers and compared to the numbers produced in single infections.

**Figure 8:**
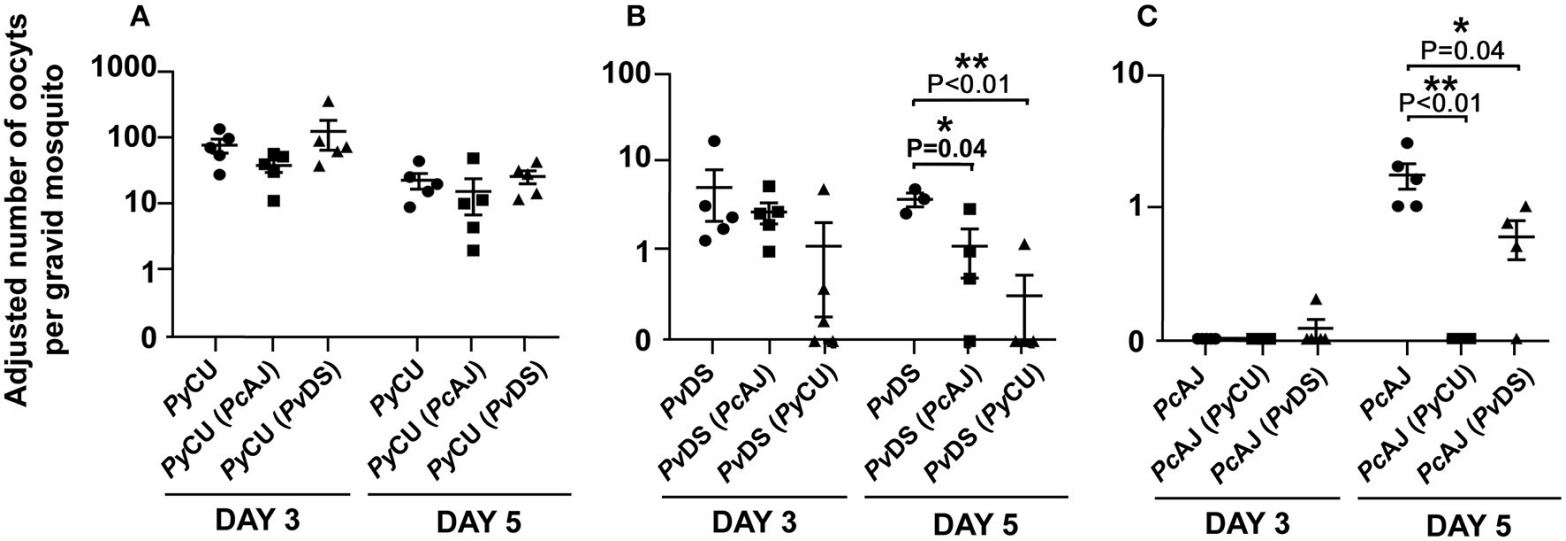
Adjusted mean number of oocysts of *Plasmodium yoelii* CU (*Py*CU), *Plasmodium vinckei* DS (*Pv*DS), and *Plasmodium chabaudi* AJ (*Pc*AJ) in single or mixed species infections per gravid mosquito. Data points represent the mean oocyst burden of mosquitoes fed on individual mice (*n* = 5 per group). There was no statistical difference between *Py*CU in single and in mixed infections with either *Pc*AJ or *Pv*DS **(A)**, *Pv*DS was suppressed when mixed with *Py*CU on day 5 PI **(B)** and *Pc*AJ was suppressed in mixed infections with *Py*CU and *Pv*DS on day 5 PI **(C)**. Detailed statistical values relating to significance are given in Supporting Table 1.

The numbers of oocysts produced by the highly infectious *Py*CU did not differ significantly between mosquitoes fed on single and mixed species infections (Figure 8A). *Pv*DS produced fewer oocysts in mixed infections with *Pc*AJ and *Py*CU than in single infections, although the effect was only statistically significant on day 5 in a mixed infection with *Py*CU (Figure 8B). *Pc*AJ also suffered a reduced transmission capacity in mixed infections, with significant reductions in oocyst numbers measured in mosquitoes fed on mixed infections with either *Pv*DS or *Py*CU on day 5, compared to those fed on single infections (Figure 8C). The transmission of *Pc*AJ to mosquitoes was completely blocked in mixed infections containing *Py*CU (Figure 8C).

## Discussion

Our results indicate that the interactions between malaria parasites co-infecting the same host can have dramatic consequences for the severity of the disease they cause. We found that when two parasite species, *P. yoelii* and *P. vinckei*, which on their own cause mild and transient disease concurrently infect the same host, the disease outcome is radically altered resulting in 100% host mortality within 15 days. This same outcome of 100% host mortality was observed in co-infections consisting of *P. yoelii* and the more virulent (but rarely lethal) *P. chabaudi*.

There are precedents for this result; Bafort (39) suggested that mixed species might increase the virulence of infections (39). Richie (9) reported that patent *P. chabaudi* infections increased their parasitaemia and duration when mixed with *P. yoelii* (9), and McGhee (40) also described a higher peak for one of the species in mixed infections (40). In contrast, Snounou et al. found a *protective* effect of mixing *P. yoelii* with *P. vinckei* or *P. chabaudi*, with less mortality in the mixed species infection groups compared to the single infection groups (34). Simlarly, Voza et al. reported that the addition of co-infecting *P. yoelii* to a *P. berghei* infection prevented the establishment of cerebral malaria, whereas this protection was not observed when co-infections with *P. vinckei* were induced (41).

More recently, Ramiro et al. (27) described increased virulence in mixed infections of *P. chabaudi* and *P. yoelii*, which they attributed to an increase in reticulocytaemia leading to enhancement of *P. yoelii* (which is reticulocyte restricted) in the mixed infections. Our results, however, are in agreement with those of Richie (9), and suggest that it is the normocyte-restricted parasite that is enhanced in mixed infections with *P. yoelii*, a result we observed both in the case of two strains of *P. chabaudi* and one of *P. vinckei*. We contend, therefore, that increasing reticulocytaemia does not explain the increase in virulence of mixed strain infections. This is supported by the fact that we also observed increased virulence (in terms of persistence of infection and decreased red blood cell density) in mixed-species infections composed of *P. chabaudi* and *P. vinckei*, both of which predominantly infect normocytes.

We found that the time at which the constituent species of a mixed-species infection were introduced to the host had a significant impact on disease outcome. Most studies on mixed malaria parasite species infections in mice introduce the constituent species contemporaneously (27, 28). For example, the parasite species combination *P. yoelii* and *P. vinckei* causes 100% mortality when introduced to mice contemporaneously. We found that when *P. yoelii* was inoculated seven days prior to the inoculation of *P. vinckei*, virulence was much reduced, although the mixed infection still caused significantly more pathology and mortality than the constituent species in single infections. Similarly, when inoculated into mice contemporaneously with *P. vinckei, P. yoelii* suffered a reduction in cumulative parasite density throughout the infection (a proxy measurement of parasite fitness), whereas *P. vinckei* was unaffected. When *P. yoelii* was introduced to mice a week before *P. vinckei*, the opposite trend was observed, with little reduction in the cumulative parasite density of *P. yoelii*, but a significant reduction in that of *P. vinckei*. We show, therefore, that it is not just the phenotypes of the constituent species of mixed infections that affect pathology and parasite fitness in combination, but also the time at which each species infects the host.

It is possible that interactions between malaria parasite species in mixed infections may be modulated through the host immune response. Molineaux et al. (42) suggested that in mixed infections of *P. falciparum* and *P. malariae*, the immune response stimulated by rising *P. falciparum* parasitaemias can inhibit *P. malariae*, but that *P. falciparum* survives longer due to its more rapid growth rate. This immune-mediated antagonism (43) agrees with observations suggesting that *P. falciparum* could reduce the prevalence of *P. malariae* (44).

There is some evidence to suggest a degree of cross-protection between malaria parasite species due to species-transcending immunity (36, 45). We wondered whether the lack of ability to control the *P. vinckei* parasitaemia toward the end of the mixed infection of *P. vinckei* and *P. yoelii* may be due to the phenomenon of “original antigenic sin” (46) rendering the acquisition of antibodies specific to *P. vinckei* sub-optimal due to the larger quantity of *P. yoelii* antigen present in the early stages of the infection. To test this, we immunized mice through exposure to and subsequent cure of a *P. yoelii* infection, and then challenged with *P. vinckei*. Contrary to the expectations of the original antigenic sin hypothesis, we observed no effect on the severity of the *P. vinckei* infection in *P. yoelii*-exposed compared to non-exposed mice.

Infection with malaria parasites is known to detrimentally affect the fitness of the infected mosquito (47). Mixed species malaria parasite infections occur in nature (4, 48), and as mixed species parasite infections caused dramatically different disease outcomes in mice, we investigated whether mosquito fitness was also affected. We measured longevity and progeny production in groups of mosquitoes fed on single and mixed species infections. In contrast to the significant alterations in pathogenicity observed in mice, there appeared to be no fitness differences between mosquitoes carrying single or mixed species infections. Linked to this, we did not observe significantly increased oocyst numbers in mosquitoes infected with mixed species, when compared to the highest-oocyst producing single constituent species, suggesting there was no significant alteration in transmission-stage investment by the species in mixed stage infections (31).

We found that the transmissibility of *P. vinckei* and *P. chabaudi* to mosquitoes was reduced in the presence of *P. yoelii* in co-infections compared to single infections. This was reflected in the lower number of oocysts of these two species in mosquitoes that had fed on mixed species infections also containing *P. yoelii* compared to those that had fed on single infections. Most significantly, transmission of *P. chabaudi* to mosquitoes was blocked completely by the presence of *P. yoelii* on day 5 of the infection. There were no reductions, however, in the numbers of *P. yoelii* oocysts. Of the three species, *P. yoelii* produces significantly higher oocyst burdens in mosquitoes than either *P. chabaudi* or *P. vinckei*, a phenomenon linked to the former species having much higher gametocyte production during the early stages of infection. One possible mechanism that may account for this observation involves gamete incompatibility; we propose that given the fact that in a mixed species infection containing *P. yoelii*, there will be significantly more *P. yoelii* microgametes than of the other species. If *P. yoelii* microgametes can recognize and attempt to fertilize the macrogametes of the second species, then a large proportion of these macrogametes will be rendered non-productive (assuming hybrids are non-viable) (49). Consistent with this theory is the fact that both *P. chabaudi* and *P. vinckei* also produce fewer oocysts when in mixed infection with each other, but much less so than when mixed with *P. yoelii*, reflecting, perhaps, the more even numbers of gametocytes produced by these two species.

A limitation of these experiments is that mosquitoes were allowed to feed on mice at only two time points, days 3 and 5 post-inoculation, rather than throughout the course of the infection. These two time points were chosen as they represent the days of maximum transmissibility of *P. yoelii* (day 3), *P. chabaudi*, and *P. vinckei* (day 5). However, these results offer only a snapshot of transmission success on these particular days, and it is possible that different outcomes would have been observed at different time-points throughout the infection.

The experiments described here were conducted using multiple strains of three species of rodent malaria parasites in 6-week old, female CBA mice. If the same experiments were to be conducted with different parasite strains and species, and in different host strains, then different outcomes might be expected. The disease progression of rodent malaria parasites is dramatically affected by mouse host strain, sex, and age (50), and it is likely that the interactions between species in mixed infections would be similarly affected. It should also be remembered that *Mus musculus* is not the natural host of the rodent malaria parasites, and neither is *Anopheles stephensi* its natural vector. It is likely that the interactions between the species tested here would result in different outcomes in their natural hosts. Furthermore, blood stage infections in mice were initiated by intravenous inoculation of infected blood, and not by the more natural route of sporozoite inoculation, another factor that may have a significant impact on the outcome of mixed species infections.

An illustration of the degree to which different outcomes may be observed in different experimental systems is given by the comparison of our results with those of Snounou et al. (34). Whereas, we observed dramatic increases in host mortality in mixed species infections containing a normocyte specialist and *P. yoelii*, Snounou et al. observed the opposite effect; with less mortality in *P. vinckei* + *P yoelii* and *P. chabaudi* + *P. yoelii* mixed species infections than in single infections of the normocyte-invaders (34). Similarly, although Ramiro et al. reported increased virulence in mixed infections in a similar manner to our observations, the mechanism for this outcome appears to differ; in our case facilitation of the normocyte invading species led to increased parasitaemia, whereas Ramiro et al. report increased growth of the reticulocyte invading species (27). In both these examples, there were differences between experiments in crucial parameters such as the sex, strain and age of the host mice used, the particular strains of malaria parasites, their passage histories, and routes of inoculation; all factors known to influence the outcome of experimental infections.

We cannot, then, make any predictions about the possible effects of mixed species human infections based on the results presented here, except to expect that there would be interactions between parasite species within the host, and that these would impact on disease outcomes.

As the world moves toward reducing the burden of malaria, the importance of mixed-species malaria infections will rise. Diagnostic techniques with improved sensitivities are revealing a greater prevalence of *P. ovale* and *P. malariae* in *P. falciparum* endemic areas than previously thought (7). Intervention strategies such as anti-vector programs, and the development and employment of new drugs and vaccine will often be more effective against one species of parasite than they will against others (51, 52). In regions where *P. falciparum* prevalence is decreasing, the prevalence of non-falciparum malaria parasite species often becomes more apparent, highlighting the importance of mixed-species infections. There is a need, therefore, to better understand how the interactions between malaria parasites species infecting the same host can impact disease progression and parasite fitness.

The experiments described here show that the disease outcomes of mixed vs. single species infections can differ and may be influenced by the phenotypic characteristics of the constituent species and the order in which they infect the host.

## Data Availability Statement

All datasets generated for this study are included in the article/Supplementary Material.

## Ethics Statement

Animal experimentation was performed in strict accordance with the Japanese Humane Treatment and Management of Animals Law (law No. 105, 1973; modified 2006), and according to the Regulations on Animal Experimentation at Nagasaki University, Japan. All procedures were approved by the Institutional Animal Research Committee of Nagasaki University.

## Author Contributions

RC and JT designed, performed, and analyzed the experiments. RC, JT, TT, and JC wrote the manuscript.

### Conflict of Interest

The authors declare that the research was conducted in the absence of any commercial or financial relationships that could be construed as a potential conflict of interest.

## Supplementary Material

Supporting Table 1 Details of statistical treatments used in the study.
